# Inflammatory Mediators of Platelet Activation: Focus on Atherosclerosis and COVID-19

**DOI:** 10.3390/ijms222011170

**Published:** 2021-10-16

**Authors:** Panagiotis Theofilis, Marios Sagris, Alexios S. Antonopoulos, Evangelos Oikonomou, Costas Tsioufis, Dimitris Tousoulis

**Affiliations:** 1 Department of Cardiology, “Hippokration” General Hospital of Athens, University of Athens Medical School, 11527 Athens, Greece; panos.theofilis@hotmail.com (P.T.); masagris1919@gmail.com (M.S.); antonopoulosal@yahoo.gr (A.S.A.); boikono@gmail.com (E.O.); ktsioufis@gmail.com (C.T.); 2 Department of Cardiology, “Sotiria” Thoracic Diseases Hospital of Athens, University of Athens Medical School, 11527 Athens, Greece

**Keywords:** inflammation, endothelium, platelet activation, atherosclerosis, COVID-19, cytokines

## Abstract

Background: Atherosclerotic cardiovascular diseases are characterized by a dysregulated inflammatory and thrombotic state, leading to devastating complications with increased morbidity and mortality rates. Summary: In this review article, we present the available evidence regarding the impact of inflammation on platelet activation in atherosclerosis. Key messages: In the context of a dysfunctional vascular endothelium, structural alterations by means of endothelial glycocalyx thinning or functional modifications through impaired NO bioavailability and increased levels of von Willebrand factor result in platelet activation. Moreover, neutrophil-derived mediators, as well as neutrophil extracellular traps formation, have been implicated in the process of platelet activation and platelet-leukocyte aggregation. The role of pro-inflammatory cytokines is also critical since their receptors are also situated in platelets while TNF-α has also been found to induce inflammatory, metabolic, and bone marrow changes. Additionally, important progress has been made towards novel concepts of the interaction between inflammation and platelet activation, such as the toll-like receptors, myeloperoxidase, and platelet factor-4. The accumulating evidence is especially important in the era of the coronavirus disease-19 pandemic, characterized by an excessive inflammatory burden leading to thrombotic complications, partially mediated by platelet activation. Lastly, recent advances in anti-inflammatory therapies point towards an anti-thrombotic effect secondary to diminished platelet activation.

## 1. Introduction

Cardiovascular diseases, the leading cause of death worldwide, are governed by a dysregulated inflammatory and thrombotic milieu, stemming from the cluster of comorbidities that encompasses them, including obesity, diabetes mellitus, and arterial hypertension. Moreover, emotional, environmental, and lifestyle aspects have been implicated in sterile inflammation and atherosclerosis development and progression as additional deleterious risk factors [1,2,3,4].

**Figure 1:**
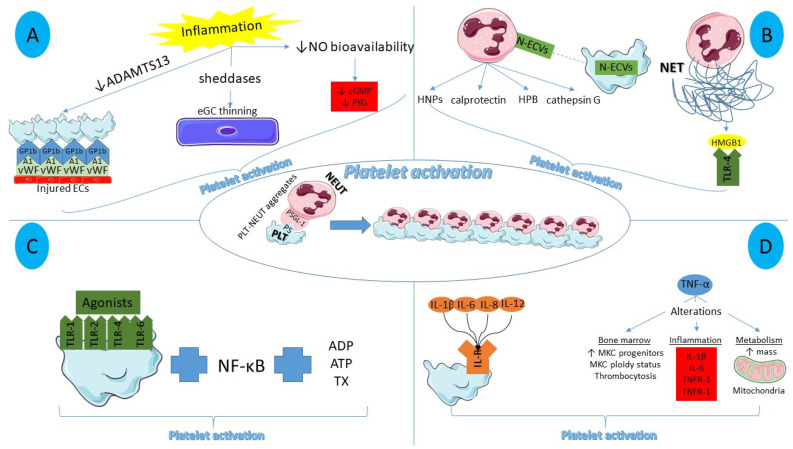
Main inflammatory mediators of platelet activation. (**A**) Endothelial dysfunction in the setting of inflammation results in increased platelet–endothelial cell interactions, thinning of endothelial glycocalyx, and reduced nitric oxide bioavailability, contributing to platelet activation. (**B**) Neutrophil-derived molecules and extracellular vesicles aid platelet activation, together with the pro-thrombotic role of NET histones via a Toll-like receptor 4-related platelet activation. (**C**) Platelet toll-like receptors cannot directly induce platelet activation but they augment it, stimulated by NF-κB, thromboxane, adenosine diphosphate, and adenosine triphosphate. (**D**) Pro-inflammatory interleukins bind to interleukin receptors situated on the platelet surface while tumor necrosis factor-alpha promotes alterations in bone marrow, inflammation, and mitochondria, collectively facilitating platelet activation. ADAMTS13: A disintegrin-like and metalloprotease with thrombospondin type-1 repeats-13, NO: nitric oxide, EC: endothelial cells, eGC: endothelial glycocalyx, cGMP: cyclic guanyl monophosphate, PKG: protein kinase G, N-ECV: neutrophil extracellular vesicles, HNP: human neutrophil peptides, HBP: heparin binding protein, NET: neutrophil extraceullar trap, HMGB1: High mobility group box 1, TLR: toll-like receptor, ADP: adenosine diphosphate, ATP: adenosine triphosphate, TX: thromboxane, IL-R: interleukin receptor, MKC: megakaryocyte, TNFR: tumor necrosis factor receptor, PS: P Selectin, PSGL-1: P Selectin glycoprotein ligand-1, NEUT: neutrophil, PLT: platelet.

Despite the progress that has been made towards the antithrombotic aspect [5,6], there is still a lot of ground to cover with regards to the management of low-grade systemic inflammation that has been proven to be a core element of the atherosclerotic process and the residual risk. The interaction of inflammation with platelets has also received scientific interest since several pro-inflammatory conditions and mediators have been associated with platelet activation. In this scoping review, we elaborate on the various inflammatory entities contributing to platelet activation (Figure 1), namely the dysfunctional endothelium, the interaction with leukocytes, and cytokines among others. Moreover, we delve into the platelet hyperactivation complicating the severe acute respiratory syndrome coronavirus-2 (SARS-CoV-2) infection and describe the current progress in the pharmacological management of thromboinflammation with a focus on platelet activation, including both currently available agents and novel molecules in the early phase of experimental development.

## 2. Inflammation and Platelet Activation

### 2.1. Endothelial Dysfunction and Platelet Activation

The vascular endothelium is a monolayer that consists of squamous cells lining the vessel wall, with a critical role in cardiovascular health. Among its well-described properties are the regulation of vascular tone, the control of cellular adhesion, the proliferation of smooth muscle cells, and the transport of macromolecules across the vessel wall [7]. Moreover, the endothelium acts in anti-thrombotic ways under physiologic situations, preventing platelet adherence and activation.

Several mechanisms have been implicated in this anti-platelet effect. Starting with the endothelial glycocalyx (eGC), its role in vascular integrity and endothelial activation has been recently characterized. The eGC is a 0.5–5 μm thick negatively charged endothelial cell cover floating into the vessel lumen that is composed of proteoglycans (syndecans, versicans), glycolipids, glycoproteins, and glycosaminoglycans (heparan sulfate, chondroitin sulfate, hyaluronan). Physiologically, the balance between the shedding of eGC components, the adsorption of circulating blood molecules, and the formation of novel constituents by endothelial cells leads to the stabilization of the eGC. However, in the setting of inflammation [8], increased shedding of eGC components, namely syndecan-1, hyaluronan, heparan sulfate, and chondroitin sulfate, is observed owing to the action of sheddases (matrix metalloproteinases, heparanase, hyaluronidase, angiopoietin 2) [9,10,11]. It has to been noted that molecules involved in thrombosis and fibrinolysis, such as plasmin and thrombin, have been found to cleave syndecan-4, thereby aiding the eGC degradation [12]. According to a recently reported study, versican might be the main eGC component that promotes an anti-platelet effect, in a dose-dependent manner [13]. Ultimately, increased platelet adhesion to the vessel wall is noted during the atherosclerotic process, owing to the eGC thinning (Figure 1A) [14], since its degradation aids the binding of leukocytes and platelets on the receptors of adhesion molecules and von Willebrand factor, which are consequently released by endothelial cells [15,16]. Additional functions of eCG have been described [17], including the binding of cytokines, the regulation of oxidative stress, and mechanotransduction.

Nitric oxide (NO) is an essential factor implicated in the regulation of platelet activation and aggregation as a physiologic inhibitor, through the activation of soluble guanylate cyclase. The resulting upregulation of intracellular cyclic guanosine monophosphate (cGMP) and protein kinase G (PKG) in platelets leads to lower intracellular calcium within platelets, an ion essential for platelet activation [18]. cGMP levels are also controlled by the platelet cGMP-specific phosphodiesterase type 5 (PDE5A) [19] but, in the absence of PKG, the effect of cGMP on intracellular Ca^2+^ is largely lost [20], depicted by increased platelet adhesion and aggregation [21]. In the presence of established cardiovascular risk factors (diabetes mellitus, arterial hypertension, dyslipidemia, smoking), the augmented oxidative stress leads to endothelial dysfunction [22]. In this setting, the low availability of NO due to its lower expression, increased degradation, and endothelial NO synthase (eNOS) uncoupling leads to enhanced peroxynitrite production, aiding the thrombotic process among others [23]. It is important to note that platelets are another source of NO as they also contain eNOS [24,25]. Even though the platelet NO production is of a lesser magnitude compared to endothelial cells [26], it is believed that its role is of greater importance as demonstrated in animal experiments [27,28].

Prostacyclin (PGI_2_) is another endothelial cell-derived molecule with a NO-synergistic effect towards lower platelet activity. By stimulating cell surface prostacyclin receptors, it provokes the activation of adenylate cyclase, the formation of cyclic adenosine monophosphate (cAMP), and the phosphorylation of protein kinase A, ultimately resulting in reduced platelet intracellular calcium [29]. It physiologically opposes the action of the prothrombotic thromboxane A2, which is also produced from endothelial cells apart from platelets [30,31]. The impaired homeostatic balance between PGI_2_ and thromboxane A2 additionally promotes a prothrombotic state in the setting of endothelial dysfunction.

Von Willebrand factor (vWF), a large multimeric glycoprotein (GP) produced mainly by the endoplasmic reticulum of endothelial cells and stored in the Weibel-Palade bodies [32], is another crucial element of hemostasis by aiding platelet adhesion through bonding of the active A1 domain with platelets via their receptor glycoprotein Ib/V/IX in areas of vascular injury [33]. In healthy states, vWF is cleaved primarily by a disintegrin-like metalloprotease with thrombospondin type-1 repeats-13 (ADAMTS13) at the Tyr1605-Met1606 site of the A2 domain [34]. However, in the presence of inflammation and oxidative stress, the diminished ADAMTS13 levels result in amplified platelet recruitment [35,36]. Interestingly, vWF unfolding at high shear rates over 5000 s^−1^ observed in areas of vascular stenosis or injury is another stimulus for platelet adhesion [37]. The role of vWF and ADAMTS13 in atherosclerosis has also been recently proven in low-density lipoprotein receptor-deficient mice crossed with mice deficient for ADAMTS13, who were then treated with recombinant ADAMTS13. Following its administration, a reduction in endothelial vWF and platelet adhesion was noted, leading to a significant decrease in aortic plaque size [38].

### 2.2. Platelet–Neutrophil Interactions in Atherosclerosis

The interaction between platelets and neutrophils has been thoroughly investigated in the setting of inflammation. Following platelet activation, the binding between their surface transmembrane protein P-selectin and its glycoprotein ligand (P-Selectin glycoprotein ligand-1, PSGL-1) on the neutrophil surface represents the main mechanism of neutrophil recruitment, with the interaction between platelet GPIb and integrin aMβ2 on neutrophils or, aided by fibrinogen, the binding of platelet integrin aIIβ3 with integrin aMβ2 as an alternative way [39]. Additionally, platelet-derived cytokines, chemokines, and microparticles have also been implicated in the recruitment and activation of neutrophils. The role of protease-activated receptor 4 in the platelet–neutrophil interactions through the secretion of dense granule contents has also been recently demonstrated experimentally [40]. Moreover, thrombospondin-1, expressed by platelets following their activation, undergoes neutrophil-mediated proteolysis leading to a lower mass molecule that is a potent inducer of platelet activation [41].

On the other hand, in the presence of atherosclerosis, the higher levels of inflammatory cytokines induce the activation of neutrophils which, in turn, overexpress adhesion molecules that promote their binding with platelets, further attenuating the processes of thromboinflammation (Figure 1B) [42]. Several other neutrophil-derived molecules have been implicated in platelet activation including human neutrophil peptides [43,44], heparin binding protein [45], and neutrophil cathepsin G [46]. Novel concepts have also been introduced with regards to neutrophil-mediated platelet activation. To begin with, the neutrophil production of calprotectin has been related to enhanced neutrophil mobilization [47] and platelet activation in experimental studies [48,49]. Moreover, the role of neutrophil cathelicidins in thromboinflammation is being investigated, with LL-37 being an important mediator of platelet activation through the GP VI receptor and downstream signaling via Src/Syk tyrosine kinases and phospholipase C [50]. Lastly, the release of extracellular vesicles from neutrophils and the subsequent entry into platelets could also result in increased thromboxane synthesis and, ultimately, platelet activation [51].

The prothrombotic role of neutrophil extracellular traps (NETs) is also of great importance in the context of atherosclerotic cardiovascular diseases [52]. Based on their composition of DNA, histones, and granular components, NETs can provoke thrombosis either through contact-dependent coagulation [53] or histone-induced platelet recruitment and activation mediated by Toll-like receptors (TLRs) [54]. High-mobility group box 1 (HMGB1) is a damage-associated molecular pattern that has been tightly associated with NET-induced thrombosis [55]. Its involvement in platelet activation has been associated with TLR4 and MyD88-dependent signaling, as well as arterial thrombi [55]. Importantly, NETs were found in abundance in coronary thrombi [56], coronary stent thrombi [57], and cerebrovascular thrombi [58], while their presence was correlated with the degree of infarct [59].

### 2.3. The Role of Myeloperoxidase and Platelet Toll-Like Receptors

The release of myeloperoxidase (MPO) by activated neutrophils in the setting of inflammation has been associated with atherosclerotic diseases, namely coronary artery disease and its most grievous manifestation, acute coronary syndrome [60,61]. MPO is implicated in alterations of the inflammatory and oxidative processes and the promotion of a stable plaque phenotype [62,63]. Moreover, recent advances have highlighted additional hazardous effects concerning platelet activation. In the study of Kolarova et al., the authors demonstrated that platelets, which are MPO-free following their release from bone marrow, are mildly activated by MPO, as evidenced by their increased production of P-selectin and platelet endothelial cell adhesion molecule-1, the rearrangement of their cytoskeleton, and the released of reactive oxygen species [64]. Interestingly, they did not detect platelet aggregation, thus signifying only partial activation which may, however, enhance the platelet reaction to other stimuli. The effect of MPO on the bioavailability of platelet-derived NO is another fact that has to be taken into account as it is also associated with increased platelet activation and, consequently, adverse cardiovascular outcomes [65].

Platelet TLRs, a cluster of 10 pattern recognition receptors responsible for the identification of molecular patterns, are also involved in the regulation of thromboinflammation (Figure 1C). Previously published studies have reported abnormalities in the levels of platelet TLR 1, 2, 4, and 6 in patients with acute coronary syndromes [66,67], while limited information is available regarding the other representatives of this family.

Starting with platelet TLR2/1, their stimulation by an agonist (Pam3CSK4), exerts pro-thrombotic and pro-inflammatory effects. However, platelet activation is not immediate following their stimulation, requiring either the non-genomic NF-κB activation [68] or the release of adenosine diphosphate or triphosphate and the concomitant release of thromboxane [69,70]. Similar to the above-mentioned complex, platelet TLR4 is incapable of inducing a profound thrombotic effect after the administration of its agonist, lipopolysaccharide, but can augment an established platelet aggregation process [71], while also requiring the presence of other surface molecules such as CD62 [72]. Agonists of platelet TLR2/6 were also deemed unable to activate platelets in physiological doses [67,73], but managed to do so at supra-physiological doses [67]. Along those lines are the observations on other platelet TLR family members such as TLR3 and TLR7 [74,75]. It can be assumed that the main role of these complexes is directed towards inflammation rather than thrombosis [76].

### 2.4. Cytokines in Platelet Activation

The role of pro-inflammatory cytokines in cardiovascular diseases events has been established with an increased risk of adverse incidents being noted when elevated [77,78]. Unsurprisingly, anti-inflammatory therapies reduced the incidence rate of adverse outcomes in high-risk individuals in the recently completed Canakinumab Antiinflammatory Thrombosis Outcome Study (CANTOS) and Colchicine Cardiovascular Outcomes Trial (COLCOT) [79,80], further cementing the pivotal role of inflammation in the natural history of atherosclerotic cardiovascular diseases.

The excess risk observed in individuals with overproduction of inflammatory cytokines could be attributed to their prothrombotic role (Figure 1D). It should be noted that platelets have receptors for interleukins (ILs), whose stimulation could lead to platelet activation [81,82]. A recent study confirmed this hypothesis, demonstrating enhanced coagulation and platelet hyper-reactivity upon exposure of whole blood from healthy volunteers to IL-1β, IL-6, IL-8, and IL-12 [83,84].

Similar results have been reported for tumor necrosis factor-α (TNF-α) which promoted platelet aggregation and activation [85], while TNF-α inhibitors abrogated platelet activation, platelet-leukocyte interactions, and tissue factor expression in patients with rheumatoid arthritis [86]. Critical insight was provided by the study of Davizon-Castillo et al. in aging-associated inflammation [87]. TNF-α was recognized by the researchers as the primary cytokine involved in platelet hyper-reactivity surrounding this process, eliciting significant alterations at the level of bone marrow (increased megakaryocyte-committed progenitor cells, megakaryocyte ploidy status, and thrombocytosis). Moreover, they reported genetic alterations in inflammatory pathways (signaling of IL-1β, IL-6, TNF receptor-1, and TNF receptor-2), in metabolic pathways, and mitochondrial function evidenced by the augmentation of mitochondrial mass. Those findings were confirmed following administration of recombinant TNF-α in young C57BL/6J mice and reversed after treatment with an anti-TNF-α antibody. It should also be noted that in the absence of functional TNF-α receptors, TNF-α overexpression had no impact on platelet activity and mitochondrial functionality. Strikingly, IL-1β treatment did not result in platelet hyper-reactivity.

### 2.5. NLRP3 Inflammasome

The nucleotide-binding oligomerization domain, leucine-rich repeat-containing receptor (NLR) family pyrin domain-containing 3 (NLRP3) inflammasome, also consisting of the adaptor apoptosis-associated speck-like protein containing a CARD (ASC) and pro-caspase-1, is well-characterized in atherosclerotic diseases. Several activating factors have been described in the NLRP3 inflammasome assembly in atherosclerosis, including hypoxia [88], NETs [89], cholesterol crystals [90], oxidized low-density lipoprotein cholesterol (LDL-C), and oscillatory shear stress among others [91].

The platelet NLRP3 inflammasome constitutes an important mediator of platelet activation, either not requiring an initial priming signal [92] or being primed in an autocrine or paracrine way in the presence of platelet IL-1 receptor [93]. Murthy et al. showed that the platelet NLRP3 inflammasome was responsible for platelet activation, aggregation, and thrombus formation [94]. Moreover, platelet activation can also induce NLRP3 inflammasome upregulation and subsequent activation of caspase-1 and IL-1β cleavage. Interestingly, they identified platelet Bruton’s tyrosine kinase (BTK) as the mediator of those prothrombotic effects. Vogel et al. shed further light towards this direction by performing BTK or NLRP3 inflammasome inhibition with ibrutinib and MCC950 respectively in sickle cell mice, noting diminished platelet aggregation and thrombus formation [95]. These findings add to the existing knowledge on the platelet activating actions of the platelet NLRP3 inflammasome via the caspase-1-induced thromboinflammatory particles [96].

### 2.6. Platelet Factor-4

Platelet factor-4 (PF4) is an important mediator of atherogenesis since it has been found in abundance in atherosclerotic plaques and its quantity is related to the severity of the atherosclerotic lesion [97]. Derived from activated platelets, PF4 is laid upon endothelial cells where it recruits monocytes and aids their differentiation into macrophages [98]. Additionally, it interacts with LDL-C by inhibiting the degradation of their receptor and facilitating the uptake of oxidized LDL-C by macrophages [98]. PF4 correlates strongly with the degree of platelet activation evidenced by the platelet-monocyte aggregates, as indicated by the study of Gremmel et al. [99], while its genetic variations are associated with alterations in platelet activation [100]. However, the binding of PF4 with endothelial cell-derived perlecan heparan sulfate chains appears to have an attenuating effect on PF4-induced platelet activation [101].

## 3. Inflammation and Platelet Activation in Coronavirus Disease-19

**Figure 2:**
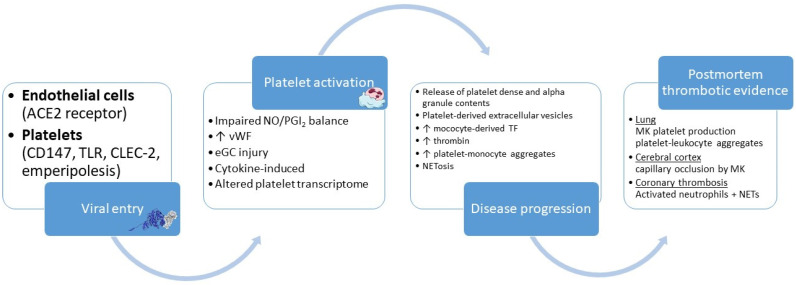
SARS-CoV-2 infection and platelet activation. Severe acute respiratory syndrome coronavirus 2 (SARS-CoV-2) enters endothelial cells through the angiotensin converting enzyme 2 (ACE2) receptor or platelets through CD147, toll-like receptors (TLR), C-type lectin-like receptor 2 (CLEC-2), or via emperipolesis. Several mechanisms are involved in the consequent platelet activation, which leads to the release of platelet granule content, the overexpression of tissue factor (TF), the formation of platelet-leukocyte aggregates, and neutrophil extracellular traps (NETs). Ultimately, the rising severity of the disease could lead to death with significant findings of thrombosis being evidenced in postmortem lung, brain, and heart studies. NO: nitric oxide, vWF: von Willebrand factor, eGC: endothelial glycocalyx, MK: megakaryocytes.

The interplay between inflammation and platelets is highly evident in the SARS-CoV-2 infection (Figure 2). The coronavirus disease-19 (COVID-19) pandemic that ensued has led to increased mortality rates owing to the endotheliitis and the cytokine storm observed in the infected patients [102], while the social distancing measures and lockdown policies resulted in restricted access to healthcare facilities potentially affecting morbidity [103,104].

Inflammation and thrombosis are the hallmark features of COVID-19. The hyperinflammatory state is believed to be the main driver of the procoagulant phenotype observed especially in critically ill COVID-19 patients [105]. Even though venous thromboembolism constitutes the predominant form of thrombotic complications, with a prevalence of approximately 15%, which is significantly higher in patients hospitalized in an intensive care unit, arterial thrombotic phenomena are not spared. According to the results of a recently reported meta-analysis, the frequency of arterial thrombosis was estimated at 3.9%, constituting mostly acute ischemic strokes and myocardial infarctions [106]. When analyzing patients with COVID-19 and an ST-elevation myocardial infarction (STEMI), Choudry et al. noted multiple culprit lesions, a higher thrombotic burden, and lowered primary percutaneous coronary intervention success rates [107]. Furthermore, a significantly higher (compared to contemporary non-COVID-19 data) stent thrombosis incidence in COVID-19 patients with STEMI has been reported [108].

Initially, the presence of thrombi in postmortem studies despite the routine anticoagulation of patients represented a surprising observation [109], indicating the potential role of platelets, which has already been characterized in infectious processes [110]. Interestingly, megakaryocytes producing platelets have been validated in pulmonary specimens of deceased patients with COVID-19, with leukocyte aggregation to platelets also being demonstrated [111], although discrepant results have also been reported, with immunothrombosis in the absence of megakaryocytes, platelets, and platelet-leukocyte aggregates [112]. In addition, cerebral cortex capillary occlusion owing to megakaryocytes has been documented as a potential cause of neurologic deterioration of patients with COVID-19 [113]. However, this finding is not specific to COVID-19 since it has also been observed in other settings of diffuse alveolar injury [114]. Important insight was provided in a recently reported study by Johnson et al. [115]. The investigators performed heart autopsies in patients who died of COVID-19 and compared those findings to a control group. Ιnitially they reported the presence of thrombosis as the most common observation in the specimens. Interestingly, they found no evidence of endothelial injury or activation in the small and large vessels, apart from a single patient with a recent myocardial infarction. However, they highlighted the role of activated neutrophils and incident NETosis in the pathophysiology of coronary thrombosis of COVID-19, in the absence of direct viral infiltration of the heart. On the contrary, autopsy on lung specimens revealed a pattern of widespread endothelial cell injury paired with viral presence as the potential pathogenetic mechanisms of thrombosis and microangiopathy [116].

It should also be noted that the thrombocytopenia frequently seen in patients with COVID-19, especially in those with the severe form of the disease [117], could be a result of the increased platelet consumption in thrombus formation [118,119] or platelet apoptosis and represents a poor prognostic indicator [120]. Consequently, the higher megakaryocyte production that is noted, also attributed to the hyper-inflammatory state and increased thrombopoietin and Janus Kinase (JAK)/Signal Transducer and Activator of Translation (STAT) signaling, may lead to increased mean platelet volume [121,122]. This platelet population has been characterized as immature and reticulated, findings that indicate increased thrombogenicity [121,123]. When considering patients with cardiovascular risk factors, who are widely characterized by platelet alterations and platelet apoptosis [124], the exposure to SARS-CoV-2 renders platelets more susceptible to activation [125].

Platelet activation in the setting of COVID-19 has been a topic of intense scientific research with several mechanisms being described. ACE2-dependent endothelial cell infection by SARS-CoV-2 has been documented early in the course of the pandemic [126] and confirmed in a recent experimental animal study of Qin et al. [127]. COVID-19 cytokine storm-induced endotheliitis is also responsible for the impaired balance of anti-thrombotic substances such as NO and PGI_2_ due to the reduced levels of arginine and the upregulation of asymmetric dimethylarginine, ultimately leading to a greater degree of platelet activation [128]. The interaction between SARS-CoV-2 and endothelial cells cannot be predicted with accuracy, since even in mild disease forms, the virus-induced vessel damage could persist for an unspecified duration [129]. The importance of vWF in platelet activation is also prevalent in COVID-19, since its levels were found significantly increased in this scenario [130].The role of eGC has also been touched upon in the setting of COVID-19, with several studies highlighting glycocalyceal damage, an increase in biomarkers associated with eGC degradation, such as syndecan-1, hyaluronic acid, and sTie-2, as well as lower levels of eGC-protective markers such as heparanase-2 [131,132]. Such changes to endothelial glycocalyx and endothelial function in general appear to be long-lasting, as described by Lampadiari et al., who followed-up patients 4 months after SARS-CoV-2 infection [133]. The prolonged endothelial dysfunction has also been confirmed by Fogarty et al. [134]. Consequently, this finding might partially explain the presence of the long COVID syndrome, which is also characterized by an increased burden of thromboembolic events [135]. Moreover, the interaction of platelets with the vascular endothelium in the setting of COVID-19 seems bidirectional, since platelet-derived myeloid-related protein 8/14 was overexpressed in SARS-CoV-2 infection and induced microvascular endothelial cell activation and dysfunction, leading to a procoagulant phenotype [136].

The presence of a cytokine storm is believed to be one of the main drivers of platelet activation, via inflammatory cytokines such as IL-6, IL-1b, and TNF-α [137]. Polymorphonuclear myeloid-derived suppressor cell expansion in COVID-19 may also stimulate platelet activation [138], since those cells have been found to overexpress mediators of L-arginine, arginase-1, and inducible NOS catabolism [139]. A direct effect of SARS-CoV-2 on megakaryocytes could also lead to hyperreactivity due to a reformed platelet transcriptome by means of altered protein ubiquitination, antigen presentation, and mitochondrial dysfunction [140], as well as changes in metabolic signaling pathways of oxidative phosphorylation and glycolysis [141]. Even though the available data point to the lack of angiotensin converting enzyme 2 (ACE2) receptor on platelets, it is believed that other receptors might be implicated in SARS-CoV-2 entry into platelets, such as CD147 [141]. Other speculative mechanisms of SARS-CoV-2 uptake by platelets may include endocytosis by TLRs, C-Type Lectin-Like Receptor 2, or megakaryocyte emperipolesis [142,143].

As a result of the above-mentioned mechanisms, activated platelets lead to the monocyte overexpression of tissue factor, responsible for thrombin generation, following the formation of platelet-monocyte aggregates, as demonstrated by Hottz et al. who incubated platelets from healthy volunteers with the plasma of patients with a severe form of COVID-19 [144]. Platelet P-selectin seems crucial for the interaction of platelets with monocytes, as shown by its positive correlation with platelet-monocyte aggregates [144]. It should also be noted that these observations were related to the severity of the illness and that the inhibition of platelet P-selectin or integrin αIIb/β3 with abciximab attenuated the process [144]. Specifically for the platelet–neutrophil interaction, the release of highly thrombogenic NETs could also be the cause of excessive thrombotic events [145], as recently demonstrated in patients with COVID-19 [146,147]. Additionally, the release of the content of dense and alpha granules from the hyperactive platelets has also been observed in patients with COVID-19, paired with increased circulating markers of platelet activation such as PF4, P-selectin, and thrombopoetin, highlighting the increased thrombotic risk present in this group of patients [148]. Lastly, extracellular vesicles have been increasingly prevalent following platelet activation [149], a finding that could also partially explain the pro-thrombotic state in SARS-CoV-2 infection [150]. Importantly, COVID-19 severity defines the characteristics of platelet-derived extracellular vesicles, since patients with the non-severe form of the disease had higher levels of phosphatidylserine-exposing platelet EV [151].

## 4. Therapeutic Implications

Inflammation and platelet activation represent crucial therapeutic targets in atherosclerotic cardiovascular diseases and COVID-19 since they are amongst the main etiologies of major adverse events. Even though antiplatelet agents have been established as the cornerstone of atherosclerotic disease management, this has not been the case for anti-inflammatory therapies despite the encouraging results following canakinumab administration in high-risk individuals with elevated levels of high sensitivity C reactive protein [79], which reduced the incidence rate of nonfatal myocardial infarction, non-fatal stroke, and cardiovascular mortality. Colchicine has also demonstrated considerable efficacy in patients with stable coronary artery disease [152] or after myocardial infarction [80], by decreasing the risk of future cardiovascular events when administered in a low dose. Colchicine’s use in cardiovascular disease prevention has been implemented in the latest guidelines of the European Society of Cardiology [153], owing to the positive results of the above mentioned clinical trials and meta-analysis [154]. Of note, low dose methotrexate failed to provide the expected clinical benefit in patients with coronary artery disease with diabetes mellitus or metabolic syndrome in the Cardiovascular Inflammation Reduction Trial (CIRT) [155]. However, inflammatory parameters were not among the inclusion criteria and, thus, the results of this trial should be interpreted with caution.

As highlighted, an interplay between inflammation and platelet activation in the context of atherosclerosis and COVID-19 is highly important and, thus, anti-inflammatory agents have been evaluated with regards to their anti-platelet potential (Table 1). Starting with TNF-α inhibitors, frequently used in autoimmune disorders with speculated cardiovascular benefits [156], their use has been associated with diminished platelet activation through a decrease in platelet–leukocyte interactions and reduced expression of P-Selection [86]. However, a study of etanercept in patients with acute myocardial infarction did not report remarkable alterations in platelet activation parameters [157], while increases in platelet aggregation were mentioned in an in-vitro study of adalimumab [158]. In the studies examining the IL-6 inhibitor tocilizumab, a reduced platelet activity was observed [159], also extending towards COVID-19 anti-thrombotic treatment [128]. Interestingly, when tocilizumab was administered to COVID-19 patients, an early and sustained increase in platelet count was observed, possibly signifying reduced platelet consumption and less thrombosis [160,161]. Finally, colchicine has undoubtedly received the most scientific attention in this aspect, with several studies highlighting its potential as an adjunctive antiplatelet approach [159,162,163,164], which might be responsible for the beneficial effects observed in patients with a recent acute coronary syndrome or chronic coronary syndromes [80,152]. Despite being studied in the setting of COVID-19, the effect of anti-inflammatory agents on established platelet activation parameters has not been assessed in this patient population and can only be speculated on.

With regards to other experimental therapeutics (Table 2), important progress has been made in NET inhibition. Li et al. noted a decrease in inflammatory cytokines paired with attenuation of platelet activation and thrombus formation following NET degradation with the inhibitor DNAse [168]. In another study, DNAse lessened ATP and ADP-induced thrombus formation in vivo, although it did not affect NET formation [169]. The DNAse inhibitor dornase alfa has recently gained attention towards COVID-19 treatment, with improvement in patient oxygenation being noted following its use in vitro and in vivo [170,171,172], while it remains to be explored whether these agents can influence clinical outcomes by halting platelet activation among other pathways. Chloramidine, a protein-arginine deiminase antagonist known for inhibiting NETosis, was also associated with reduced arterial thrombosis in vivo [173]. Moving to TLR4 inhibition with eritoran, Clark et al. demonstrated its potential in interfering with platelet activation, as shown by the reduced platelet-leukocyte aggregates and platelet-associated NET formation [174]. Resveratrol was also found to possess anti-TLR4 properties, leading to the attenuation of thrombin receptor activated peptide (TRAP)- and collagen-induced platelet aggregation as well as the reduction of CD40L [175]. It is critical to note that currently available antiplatelet agents appear to have minimal effect on platelet TLR4 [176]. Moreover, novel aptamers acting as inhibitors of vWF have been developed (BT200, DTRI-031), which attenuate the platelet-activating properties of vWF in a dose-dependent manner [177,178]. Lastly, the role of NLRP3 inflammasome inhibition is gathering scientific attention, with either direct inhibitors (MCC950) or BTK inhibitors (acalabrutinib, ibrutinib) demonstrating efficacy in attenuating platelet activation, aggregation, and thrombus formation in experimental studies [179,180,181,182].

## 5. Conclusions

The interaction of platelets with the vascular endothelium, the immune cells, and other inflammatory mediators appears to be a central component of thromboinflammation, which adversely affects cardiovascular health by promoting atherogenesis. Moreover, platelet activation as a result of the cytokine storm induced by SARS-CoV-2 infection might be a crucial moderator of the thrombotic complications reported in COVID-19 patients. Ultimately, according to the results of preclinical and clinical studies, anti-inflammatory therapies could represent a reasonable therapeutic option in the reduction of residual cardiovascular risk in the setting of atherosclerotic diseases and COVID-19.

## Figures and Tables

**Table 1:** Studies evaluating the effect of the available anti-inflammatory agents on platelet activation parameters.

Study	Agent	Population	Findings
Di Sabatino et al. [165]	Infliximab	IBD	↓ TX biosynthesis ↓ sCD40L
Manfredi et al. [86]	Anti-TNF-α	RA	↓ platelet-leukocyte and platelet-monocyte aggregates ↓ P-selectin
Menchen et al. [166]	Infliximab	CD	↓ sCD40L Non-significant reduction of the enhanced binding of FITC–fibrinogen
Padfield et al. [157]	Etanercept	AMI	Non-significant effects on platelet activation parameters
Nielsen et al. [158]	Adalimumab	In vitro	↑ TRAP-induced platelet aggregation ≥20%
	Tocilizumab		↓ ADP- and collagen-induced platelet aggregation ≥20%
Canzano et al. [128]	Tocilizumab	COVID-19 (in vitro)	↓ P-Selectin ↓ Platelet-granulocyte and platelet-neutrophil aggregates
Shah et al. [159]	Colchicine	Healthy	↓ P-Selectin and PAC-1 ↓ Platelet-granulocyte and platelet-neutrophil aggregates No effect on homotypic platelet aggregation
Cirillo et al. [162]	Colchicine	CAD	↓ TRAP-induced platelet aggregation
Cimmino [163]	Colchicine	CAD (in vitro)	ADP/Collagen/TRAP-induced platelet aggregation due to cytoskeleton reorganization
Pennings [164]	Colchicine	Healthy (in vitro)	↓ P-Selectin ↓ collagen-induced platelet aggregation
DeSena et al. [167]	Canakinumab	Case of epilepsy	Downregulation of genes associated with platelet activation

TX: thromboxane, sCD40L: soluble cluster of differentiation 40 ligand, FITC: Fluorescein isothiocyanate, AMI: acute myocardial infarction, TRAP: Thrombin Activating Receptor Peptide, ADP: adenosine diphosphate. ↓ indicates decreased and ↑ indicates increased

**Table 2:** Studies evaluating the effect of the experimental anti-inflammatory agents on thromboinflammation.

Study	Agent	Target	Findings
Li et al. [168]	DNAse	NETs	↓ platelet activation ↓ thrombus formation
Carminita et al. [169]	DNAse	NETs	↓ ATP and ADP-induced thrombus formation
Novotny et al. [173]	Chloramidine	NETs	↓ arterial thrombus formation
Clark et al. [174]	Eritoran	TLR4	↓ platelet-leukocyte aggregation ↓ platelet-mediated neutrophil degranulation
Sun et al. [175]	Resveratrol	TLR4	↓ TRAP- and collagen-induced platelet aggregation ↓ platelet adhesion and secretion ↓CD40L and platelet factor-4
Kovacevic et al. [177]	BT200	vWF	Inhibition of high shear rate- and ristocetin-induced platelet aggregation
Nimjee et al. [178]	DTRI-031	vWF	Inhibition of platelet aggregation and thrombosis
Dobie et al. [180]	Ibrutinib	BTK	ADAM17-mediated shedding of GPIbα and GPIX in vitro and of GPIbα in vivo
Ninomoto et al. [181]	Ibrutinib	BTK	↓ collagen-induced platelet aggregation
Nicolson et al. [179]	Ibrutinib	BTK	↓ rhodocytin-induced platelet aggregation and granule secretion
	Acalabrutinib		↓ rhodocytin- and podoplanin-induced platelet aggregation Blocks CLEC-2-mediated platelet activation and granule secretion
Cornelius et al. [182]	MCC950	NLRP3 inflammasome	↓ platelet activation

NET: neutrophil extracellular trap, ATP: adenosine triphosphate, ADP: adenosine diphosphate, FITC: Fluorescein isothiocyanate, CD40L: cluster of differentiation 40 ligand, vWF: von Willenbrand factor, BTK: Bruton’s tyrosine kinase, GP: glycoprotein. ↓ indicates decreased.

## Data Availability

Not applicable.
